# Hair regrowth in alopecia areata patients following Stem Cell Educator therapy

**DOI:** 10.1186/s12916-015-0331-6

**Published:** 2015-04-20

**Authors:** Yanjia Li, Baoyong Yan, Hepeng Wang, Heng Li, Quanhai Li, Dong Zhao, Yana Chen, Ye Zhang, Wenxia Li, Jun Zhang, Shanfeng Wang, Jie Shen, Yunxiang Li, Edward Guindi, Yong Zhao

**Affiliations:** Department of Dermatology, The First Hospital of Hebei Medical University, Shijiazhuang, Hebei 050031 P.R. China; Cell Therapy Center, The First Hospital of Hebei Medical University, Shijiazhuang, Hebei 050031 P.R. China; Department of Neurology, Jinan Central Hospital, Shandong University, Jinan, Shandong 250013 P.R. China; Department of Pathology, Jinan Central Hospital, Shandong University, Jinan, Shandong 250013 P.R. China; Department of Obstetrics, Jinan Central Hospital, Shandong University, Jinan, Shandong 250013 P.R. China; Tianhe Stem Cell Biotechnologies Inc., Jinan, Shandong 250055 P.R. China; CORD:USE Cord Blood Bank, Orlando, FL 32810 USA; Department of Research, Hackensack University Medical Center, 40 Prospect Avenue, Hackensack, NJ 07601 USA

**Keywords:** Alopecia areata, Autoimmune, Stem Cell Educator, Immune modulation, Hair regrowth

## Abstract

**Background:**

Alopecia areata (AA) is one of the most common autoimmune diseases and targets the hair follicles, with high impact on the quality of life and self-esteem of patients due to hair loss. Clinical management and outcomes are challenged by current limited immunosuppressive and immunomodulating regimens.

**Methods:**

We have developed a Stem Cell Educator therapy in which a patient’s blood is circulated through a closed-loop system that separates mononuclear cells from the whole blood, allows the cells to briefly interact with adherent human cord blood-derived multipotent stem cells (CB-SC), and returns the “educated” autologous cells to the patient’s circulation. In an open-label, phase 1/phase 2 study, patients (N = 9) with severe AA received one treatment with the Stem Cell Educator therapy. The median age was 20 years (median alopecic duration, 5 years).

**Results:**

Clinical data demonstrated that patients with severe AA achieved improved hair regrowth and quality of life after receiving Stem Cell Educator therapy. Flow cytometry revealed the up-regulation of Th2 cytokines and restoration of balancing Th1/Th2/Th3 cytokine production in the peripheral blood of AA subjects. Immunohistochemistry indicated the formation of a “ring of transforming growth factor beta 1 (TGF-β1)” around the hair follicles, leading to the restoration of immune privilege of hair follicles and the protection of newly generated hair follicles against autoimmune destruction. Mechanistic studies revealed that co-culture with CB-SC may up-regulate the expression of coinhibitory molecules B and T lymphocyte attenuator (BTLA) and programmed death-1 receptor (PD-1) on CD8β^+^NKG2D^+^ effector T cells and suppress their proliferation via herpesvirus entry mediator (HVEM) ligands and programmed death-1 ligand (PD-L1) on CB-SCs.

**Conclusions:**

Current clinical data demonstrated the safety and efficacy of the Stem Cell Educator therapy for the treatment of AA. This innovative approach produced lasting improvement in hair regrowth in subjects with moderate or severe AA.

**Trial registration:**

ClinicalTrials.gov, NCT01673789, 21 August 2012

## Background

Alopecia areata (AA) is one of the most common T cell-mediated autoimmune skin diseases, leading to chronic and relapsing hair loss. AA affects both children and adults of all ages and on hairs of all colors, with a prevalence rate at 2% of the overall population without gender predilection [1]. Clinical evidence supports a high prevalence of comorbid autoimmune conditions among individuals with AA, such as thyroid disease, type 1 diabetes mellitus, inflammatory bowel disease, and systemic lupus erythematosus [1,2]. The quality of life in AA patients has been significantly affected by the disappointing outcomes, side effects, and relapses with current conventional therapies, including topical and systematic applications of immunosuppressive regimens (such as corticosteroids and cyclosporine) or immune modulators (e.g., dithranol and diphenylcyclopropenone (DPCP)) [1,3]. To overcome these challenges, an innovative and translational technology is necessary to advance the current management of AA. Ideally, this clinical approach should address multiple or all of the underlying causes of autoimmunity in AA. However, similar to all other autoimmune diseases, possible triggers for autoimmunity in AA include genetic, epigenetic, physical, emotional, social, and environmental factors. These complicated factors may act independently or jointly to break down the “immune privilege” of hair follicles through different molecular and cellular mechanisms, resulting in the autoimmune destruction of hair follicles by multiple immune cells, such as CD4^+^ and/or CD8^+^ T cells and natural killer (NK) cells [1,4-7]. Thus, a comprehensive approach is needed to fundamentally restore the immune privilege of hair follicles and address these multiple immune dysfunctions resulting from a variety of etiological causes.

Hair follicles are normally immune privileged sites, similar to other organ tissue systems, such as the brain, eye, and testis, and they contribute to the regulation of homeostasis through the neuroendocrine-immune network [8,9]. Under physiological conditions, the maintenance of the immune privilege status may include the following potential mechanisms: a low expression or absence of major histocompatibility complex (MHC) class I antigens and MHC class I chain-related A (MICA) molecules; a presence of functionally impaired Langerhans cells; and local expression of potent immunosuppressants (for example, transforming growth factor beta 1 (TGF-β1) and α-melanocyte stimulating hormone (MSH)) [5-7,10]. It is well recognized that collapse of hair follicle immune privilege leads to the onset of AA [7,11-14]. Due to the complexity of AA-related autoimmune responses and the similarity with other autoimmune diseases, clinical therapies and trials that only target one or a few components of the autoimmune responses are likely to fail, as has been observed in recent clinical trials for type 1 diabetes [15-17]. Successful immune therapies will likely restore the immune balance and peripheral tolerance by a comprehensive modulation within the entire human immune system.

We have previously characterized a novel type of stem cell from human umbilical cord blood, designated a cord blood-derived multipotent stem cell (CB-SC) [18,19]. CB-SCs are phenotypically and functionally different from other types of stem cells [20], including hematopoietic stem cells (HSCs), mesenchymal stem cells (MSCs), endothelial progenitor cells (EPCs), and monocyte-derived stem cells [21,22]. Preclinical work demonstrated the immune modulation capability of CB-SCs in autoimmune-caused diabetic non-obese (NOD) mice [23] as well as with autoreactive human T cells from type 1 diabetic patients [19]. Recently, we reported on the development of the Stem Cell Educator therapy utilizing cultured CB-SCs in clinical trials for both type 1 and type 2 diabetes [20,24,25]. Clinical data demonstrated that a single treatment with the Stem Cell Educator provided lasting reversal of autoimmunity and a rebalance of immune responses that allowed regeneration of islet β cells and improvement of metabolic control in subjects with long-standing type 1 diabetes [20,24]. Additionally, a phase 1/2 clinical study demonstrated that Stem Cell Educator therapy can control immune dysfunction and restore the immune balance through the modulation of monocytes/macrophages, leading to a long-lasting improvement of insulin sensitivity and metabolic control in long-standing type 2 diabetic patients [25]. The combined preclinical and early clinical data [19,20,23-26] raise the intriguing possibility that the Stem Cell Educator therapy may also be useful in overcoming the autoimmunity involved in AA. Here, we explore the therapeutic potential of Stem Cell Educator therapy in AA subjects.

## Methods

### Cell proliferation and *ex vivo* co-cultures

Human buffy coat blood units were purchased from the Blood Center of New Jersey (East Orange, NJ, USA). Human peripheral blood-derived mononuclear cells (PBMCs) were harvested as previously described [24,25]. The PBMCs were stimulated for 5 days with Dynabeads coupled with anti-CD3, anti-CD28, and anti-CD137 antibodies (Life Technologies, Grand Island, NY, USA) in the presence of 50 U/ml recombinant human IL-2 (rIL-2) and 5 ng/ml recombinant human IL-7 (rIL-7) (R&D Systems, Minneapolis, MN), and incubated at 37°C, in 8% CO_2_. The proliferation of lymphocytes was stained and analyzed with CellTrace™ CFSE Cell Proliferation kit (Life Technologies) following the manufacturer’s instructions. The Dynabeads were removed for flow cytometry by using DynaMag-15 (Life Technologies) according to the manufacturer’s instructions.

To perform *ex vivo* studies, human cord blood units were provided by the CORD:USE Cord Blood Bank (Orlando, FL, USA). All cord blood samples were screened for alanine aminotransferase (ALT) and pathogenic antigen antibodies (including anti-HCV, anti-HBsAg, anti-HIV, anti-Syphilis, anti-Chlamydia, and anti-Gonorrhea Abs), and only pathogen-free cord blood units were used for isolating CB-SCs. Human cord blood-derived stem cells (CB-SCs) were generated as previously described [24,25] with the following modifications. Cord blood mononuclear cells were plated in serum-free culture medium (Lonza, Walkersville, MD, USA) and incubated at 37°C, in 8% CO_2_. After 2 to 3 weeks, CB-SCs growing at 80-90% confluence were prepared for co-culture with allogeneic lymphocytes.

### Flow cytometry

Flow cytometric analyses were performed as previously described [23]. Cells were incubated with mouse anti-human monoclonal antibodies (mAb; Beckman Coulter, Brea, CA, USA), including APC-Alexa Fluor 750-conjugated anti-CD4 and anti-CD66b, Krome Orange-conjugated anti-CD8α, anti-CD14, and anti-CD19, phycoerythrin (PE)-conjugated anti-CD8β and anti-CD123, APC-conjugated anti-CD11c, phycoerythrin-Cy7 (PE-Cy7)-conjugated anti-BTLA, R Phycoerythrin-Cyanine 5.5 (PC5.5)-conjugated anti-PD-1, and FITC-conjugated anti-HLA-DR. FITC-conjugated mouse anti-human CD45 mAb was purchased from BD Biosciences (San Jose, CA, USA). PE-conjugated mouse anti-human CD270 (HVEM) mAb was purchased from BioLegend (San Diego, CA, USA). Alexa Fluor 647-conjugated rat anti-human Oct 3/4 mAb was purchased from eBioscience (San Diego, CA, USA). Cells were stained for 30 min at room temperature and then washed with PBS prior to flow analysis. Isotype-matched mouse anti-human IgG antibodies (Beckman Coulter) served as a negative control for all fluorescein-conjugated IgG mAb. For intracellular staining, cells were fixed and permeabilized using a PerFix-nc kit (Beckman Coulter). After staining, cells were collected and analyzed using a Gallios Flow Cytometer (Beckman Coulter), equipped with 3 lasers (488 nm blue, 638 red, and 405 violet lasers) for the concurrent reading of up to 10 colors. The final data were analyzed using the Kaluza flow cytometry analysis software (Beckman Coulter).

### Patients

The AA subjects were consecutive patients receiving care through the Department of Dermatology at the First Hospital of Hebei Medical University (Shijiazhuang, Hebei, China) who were enrolled in a phase 1/phase 2, open-label clinical trial conducted from 29 August 2012 through 31 July 2014. With oversight from a planning committee, the principal investigator designed the trial and received ethical approval for the clinical treatment protocol and consent from the First Hospital of Hebei Medical University (Shijiazhuang, Hebei, China). Helsinki protocols were followed. Participants and their parents provided written informed consent to participate in this study, and for the publication of images and details related to the individual participants. Thirty subjects were approached for the study. The trial was conducted with nine subjects with established AA (mean alopecic duration of 5 years) (Table 1). Patients were qualified for enrollment if they met the Alopecia Areata Investigational Assessment Guidelines of the National Alopecia Areata Foundation (NAAF). All subjects receiving Stem Cell Educator therapy had been treated with current standard therapy, but the treatment failed. With at least a 3-month washout period, subjects received one treatment with the Stem Cell Educator therapy. Key exclusion criteria included: clinical fever; clinically significant liver, kidney, or heart disease; pregnancy; immunosuppressive medication; viral and bacterial diseases; or diseases associated with immunodeficiency; or any other clinically significant, coexisting conditions.

**Table 1:** **Characteristics of the AA subjects before treatment**

**Patient number**	**Age**	**Gender**	**Marriage**	**Duration (year)**	**Diagnosis**
1	21	M	No	3	Patchy alopecia Areata
2	26	F	Yes	15	Patchy alopecia Areata
3	12	F	No	6	Patchy alopecia Areata
4	20	F	No	0.8	Alopecia totalis
5	18	F	No	0.2	Alopecia totalis
6	24	M	No	2	Alopecia universalis
7	17	M	No	16	Alopecia universalis
8	17	M	No	3	Alopecia universalis
9	26	F	Yes	0.2	Alopecia universalis
**Mean (SD)**	**20 (5)**			**5 (6)**	

### Stem Cell Educator therapy and follow-up

**Figure 1:**
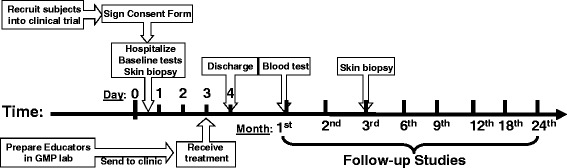
Diagram of Stem Cell Educator therapy for the treatment and follow-up studies.

Nine participants received a single treatment with the Stem Cell Educator (Tianhe Stem Cell Biotechnologies®, Jinan, China) and follow-up studies, as described in following diagram (Figure 1).

The preparation of CB-SC cultures and Stem Cell Educators was performed as previously described [24]. Briefly, human cord blood units derived from healthy allogeneic donors were obtained from Maternal and Child Health Hospital (Jinan, Shandong, China). All cord blood samples were screened for alanine aminotransferase (ALT) and pathogenic antigen antibodies (including anti-HCV, anti-HBsAg, anti-HIV, and anti-Syphilis Abs), and only pathogen-free cord blood units were used for isolating CB-SCs. Human CB-SCs were generated as previously described with the following modifications [18,23]. Cord blood mononuclear cells were plated in serum-free culture medium (Lonza, Walkersville, MD) and incubated at 37°C, in 8% CO_2_. After 2 to 3 weeks, CB-SCs growing at 90% confluence were prepared for clinical trial. The endotoxin level was < 0.05 EU/ml. One Educator device was generated from one cord blood unit, and used for one subject.

For Stem Cell Educator therapy, a 16-gauge IV needle was placed in the left (or right) median cubital vein, and the patient’s blood was passed through a Blood Cell Separator MCS+ (Haemonetics®, Braintree, MA, USA) for 6 to 7 hours to isolate mononuclear cells in accordance with the manufacturer’s recommended protocol. The collected mononuclear cells were transferred into the device for exposure to allogeneic CB-SCs, and other blood components were automatically returned to the patient. In the Stem Cell Educator, mononuclear cells separated from a patient’s blood are slowly passed through the stacked discs of material with adherent CB-SCs. After 2 to 3 hours in the device, CB-SC-treated mononuclear cells were returned to the patient’s circulation via a dorsal vein in the hand with physiological saline (2 to 3 ml/min). Approximately 10,000 ml of blood was processed during the procedure resulting in approximately two repeated educations for the lymphocyte fraction. The whole process took 8 to 9 hours. During the apheresis, some of the patients received oral calcium gluconate solution (10%, 10 ml) to ease the tingling of the lips or toes, due to the hypocalcemia caused by the citrate anticoagulant. They did not receive any other medications (such as antibiotics) during the course of treatment. Patients were hospitalized for one day to monitor temperature and conduct blood count tests for adverse reactions following treatment. Follow-up visits were scheduled 4, 12, 24, 40, 56, 84, and 112 weeks after treatment for clinical assessments and laboratory tests. Skin biopsies of scalps were performed before the treatment and at 12 weeks post-treatment. The time points for follow-up studies were similar for all subjects. Previous work demonstrated that participants receiving sham therapy failed to show changes in immune modulation [24]. Thus, the main outcome measures in the current trial were changes in hair growth and immune markers between baseline and follow-up.

### Study end points

The primary study end points were feasibility and safety of the Stem Cell Educator therapy through 24 weeks post-treatment and preliminary evaluation of the efficacy of the therapy for improving hair growth in AA subjects. The secondary study end point was preliminary evidence for efficacy of the therapy in modulating autoimmunity by flow cytometry [24,25]. Baseline blood samples and scalp tissues by biopsy were collected prior to Stem Cell Educator therapy.

### Immunohistochemistry and histology

Biopsied scalp tissues were fixed in 10% formaldehyde, embedded in paraffin, and processed for hematoxylin and eosin (H&E) staining. To determine phenotypes of infiltrated leukocytes and released cytokines, cryosections of frozen tissues were used. Cryosections (5 μm thick) of frozen tissues from AA subjects before and post-treatment with Stem Cell Educator therapy were prepared using a Leica CM1850 cryostat [23]. Cryosections were immunostained with different mAbs including FITC-conjugated anti-CD1c, anti-CD11b, anti-CD14, anti-CD83, anti-DEC205 (eBioscience), anti-TGF-β1 (BioLegend), PE-conjugated anti-TGF-β1 (BioLegend), and Alexa Fluor 488-conjugated anti-human Foxp3 (eBioscience), followed by imaging with an Olympus IX71 inverted microscope. The fluorescence intensity was measured by using the ImageJ 1.46 software.

### Statistical analysis

An intention-to-treat approach was used, with nine patients undergoing Stem Cell Educator therapy. All patients were included in safety analyses. The primary efficacy end points were hair regrowth and the change in immune markers between baseline and follow-up. Statistical analyses of data were performed using the two-tailed Student’s *t*-test to determine statistical significance. Values were given as mean ± SD (standard deviation).

## Results

### Suppressed proliferation of antigen-specific T cells by co-culture with CB-SCs

**Figure 2:**
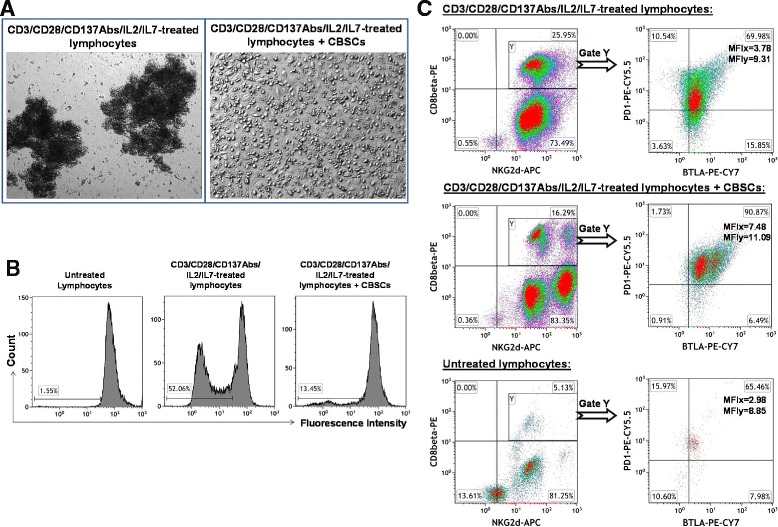
Ex vivo studies of the immune modulation of CB-SCs on T cells. **(A)** Phase contrast microscopy shows the formation of cell clusters in human peripheral blood-derived lymphocytes that were activated with Dynabeads coupled with anti-CD3, anti-CD28, and anti-CD137 antibodies, 50 U/ml rIL-2, and 5 ng/ml rIL-7 for 5 days, in absence (left panel) and presence (right panel) of CB-SCs. Co-culture with lymphocytes (top right panel) served as control. Original magnification, × 100. **(B)** Cell proliferation was analyzed with CellTrace™ CFSE Cell Proliferation Kit. Untreated lymphocytes (left panel) served as control. **(C)** Multi-color flow cytometry on CD8^+^NKG2D^+^ T cells. The gated CD8^+^NKG2D^+^ T cells were further analyzed for the expression of coinhibitory molecules BTLA and PD-1. Isotype-matched IgG Abs served as control for flow cytometry. Mean fluorescence intensity (MFI) was presented for CD8^+^NKG2D^+^BTLA^+^PD-1^+^ T cells. Flow cytometry dot plots and the percentage of each population were representative of three independent experiments with similar results.

The expansion of antigen-specific autoreactive T cells is the critical step leading to the destruction of tissues in autoimmune diseases. Recently, mouse and human data have demonstrated that CD8^+^NKG2D^+^ effector T cells function as a key mediator in the pathogenesis of AA [27]. To explore the therapeutic potential of CB-SC in AA, CD8^+^NKG2D^+^ effector T cells from human peripheral blood mononuclear cells (PBMC) were activated and expanded with Dynabeads coupled with anti-CD3, anti-CD28, and anti-CD137 mAb in the presence of IL-2 and IL-7. After *ex vivo* expansion with this mAb combination for 5 days, there were large numbers of cell clusters with different sizes floating in the supernatant (Figure 2A, left panel), suggestive of significant cell proliferation. However, this phenomenon was not evident in the presence of CB-SCs (Figure 2A, right panel). Flow cytometry revealed that 52% of lymphocytes proliferated in response to costimulation with this combination of mAb molecules and growth factors (Figure 2B, middle panel). By contrast, there were only 13% of lymphocytes proliferating after co-culture with CB-SCs (Figure 2B, right panel). Triple color staining demonstrated that 25% of CD8^+^NKG2D^+^ T cells were proliferated upon costimulation in the absence of CB-SCs. Notably, the percentage of proliferating CD8^+^NKG2D^+^ T cells was reduced to 5% following co-culture with CB-SCs. Further multi-color flow cytometry indicated that the percentage of CD8^+^NKG2D^+^ T cells was decreased from 25.6% ± 0.43% to 13.87% ± 3.43% in the presence of CB-SCs (Figure 2C, left panels) *P =* 0.04). Additionally, we examined the expression of coinhibitory molecules on CD8^+^NKG2D^+^ T cells, such as BTLA (B and T lymphocyte attenuator) and PD-1 (programmed death-1 receptor). Results confirmed that co-culture with CB-SCs increased the percentage of CD8^+^NKG2D^+^BTLA^+^PD-1^+^ T cells from 69% to 91%. Their mean fluorescence intensity (MFI) also increased after co-culture with CB-SCs (Figure 2C, right panels). These data demonstrated that CB-SCs could markedly suppress the proliferation of CD8^+^NKG2D^+^ T cells and up-regulate the expression of coinhibitory molecules on those cells. This finding supports the clinical-translational potential of CB-SCs in AA subjects.

### Expression of herpesvirus entry mediator (HVEM, CD270) on CB-SCs

**Figure 3:**
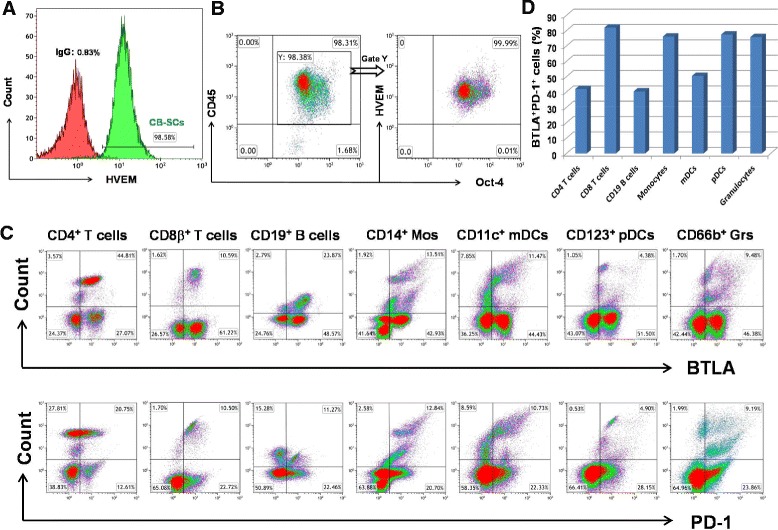
Flow cytometry analysis. **(A)** Expression of HVEM on CB-SCs. Isotype-matched IgG served as control. **(B)** Expression of HVEM on the gated CD45^+^Oct3/4^+^ CB-SCs. **(C)** Expression of BTLA and PD-1 on peripheral blood-derived immune cells. **(D)** The percentage of BTLA^+^ PD-1^+^ cells in each subpopulation. Each population was gated using the specific surface markers as described in Methods.

Human CB-SCs mediate immune modulation through the release of soluble factors (for example, nitric oxide and TGF-β1) and the expression of surface molecules such as PD-L1 (programmed death-1 ligand) [19,20,28]. To investigate additional potential molecular mechanisms underlying the immune modulation, we also found that CB-SCs strongly displayed the surface molecule HVEM (Figure 3A), the ligand of BTLA. Triple color staining confirmed the co-expression of HVEM on CB-SCs positive with molecular markers of leukocyte common antigen CD45^+^ and embryonic transcription factor Oct3/4^+^ (Figure 3B).To further substantiate that HVEM may be a mechanism of immune modulation by CB-SCs, flow cytometry also demonstrated the expression of BTLA and PD-1 on most immune cells, including CD4^+^ and CD8β^+^ T cells, CD19^+^ B cells, CD14^+^ monocytes, CD11c^+^ myeloid dendritic cells (mDCs), CD123^+^ plasmacytoid dendritic cells (pDCs), and CD66b^+^ granulocytes (Figure 3C). There were about 40-80% BTLA^+^PD-1^+^ cells in each subpopulation of immune cells (Figure 3D). These data suggest that CB-SCs may display a broad spectrum of modulatory capacity on immune cells via HVEM/BTLA and PD-L1/PD-1 signaling pathways.

### Hair regrowth in alopecia areata subjects

**Figure 4:**
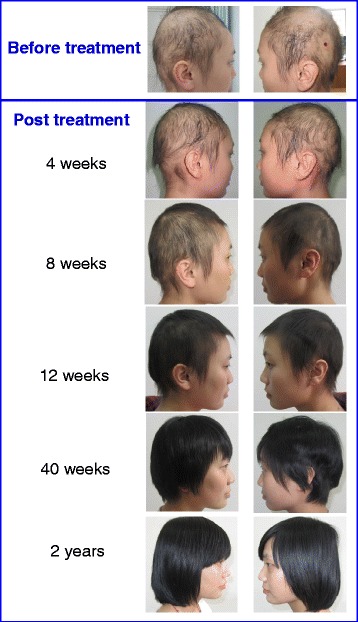
Regrowth of hair following Stem Cell Educator therapy. A subject with severe AA (patient 4 in Table 1) achieved complete hair regrowth at 12 weeks follow-up after receiving Stem Cell Educator therapy and maintained regrowth through the last follow-up (2 years).

Nine AA subjects received one treatment with Stem Cell Educator therapy and completed the study. All patients tolerated the procedure well, without any significant adverse events during the course of treatment. Their baseline clinical characteristics are described in Table 1. No participants experienced any significant adverse events during the course of treatment and the 2-year follow-up period. At 4 weeks post-treatment with Stem Cell Educator therapy, there was hair regrowth in subjects with patchy AA and alopecia totalis (Figure 4). There were short vellus hairs on the scalp of patients with AA universalis (3/4) at the 12-week follow-up. Two participants (one having alopecia totalis and another having multiple patches of AA) achieved complete hair regrowth at 12 weeks and 16 weeks, post-treatment, respectively, and remained completely recovered with no relapse after 2 years (Figure 4). Patients (3/4) with alopecia universalis exhibited regrowth of eyebrows and eyelashes at the 12-week follow-up. Notably, the regrowth of eyebrows and a mustache occurred in a 17-year-old boy affected by severe alopecia universalis since he was 1 year old. Additionally, one of nine subjects with nail pitting also improved, as indicated by the reduction of the number and the cavity of nail pitting at 4 weeks after receiving Stem Cell Educator therapy. All of these improvements were maintained throughout the final follow-up at 2 years. Of nine AA subjects, only one participant with alopecia universalis failed to show a response to the Stem Cell Educator therapy, possibly due to a previous long-term therapy with oral prednisone. Overall, the proof-of-concept data demonstrated the therapeutic potential of Stem Cell Educator therapy for the treatment of AA subjects.

### Systematic immune modulation after receiving Stem Cell Educator therapy

**Figure 5:**
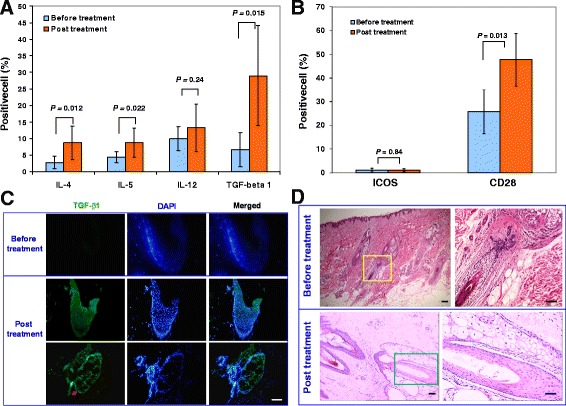
Immune modulation of Stem Cell Educator therapy. Patient lymphocytes were isolated from peripheral blood by Ficoll-Hypaque technique (γ = 1.077) for flow cytometric analyses in AA patients at baseline and 4 weeks after Stem Cell Educator therapy. Isotype-matched IgG served as control. Histologic examination of alopecic skin **(C** and **D)**. **(A)** Flow analysis of intracellular cytokines demonstrating differential effects on key interleukins at 4 weeks post-treatment. **(B)** Flow analysis of costimulating molecules demonstrating an increase of CD28 expression at 4 weeks post-treatment. Data are representative of preparations from all AA participants (n = 9) who received Stem Cell Educator therapy. **(C)** Fresh skin tissues were collected from the scalps via biopsy for immunohistochemistry testing in participants with alopecia totalis before treatment and 12 weeks after receiving Stem Cell Educator therapy. TGF-β1 staining surrounds a hair follicle of AA participants after receiving Stem Cell Educator therapy, with vertical section of hair follicle (top panels) and horizontal section of hair follicle. Isotype-matched mouse IgG_1_ served as a negative control for TGF-β1 immunostaining in a serial hair follicle section. Representative images were obtained from five experiments. Scale bar, 25 μm. **(D)** H&E staining of scalp tissues. Scale bar, 25 μm.

To explore the immune modulation of Stem Cell Educator therapy in AA subjects, we examined changes in the percentage of regulatory T cells (Treg) in their peripheral blood by using the specific intracellular marker, FoxP3. Flow cytometry revealed that the percentage of FoxP3^+^ Treg at 4 weeks was unchanged from the baseline (1.69% ± 1.02 versus 1.38% ± 0.85, *P* = 0.49). This result suggested that the immune modulation via Stem Cell Educator therapy in AA subjects may act through a different mechanism than induction of Treg. TGF-β1, one of the best-characterized cytokines contributing to the induction of peripheral immune tolerance [29], also plays a crucial role in modulating the normal cycling of hair follicles [30]. Flow cytometry demonstrated a marked increase in TGF-β1 expression by blood mononuclear cells 4 weeks after receiving Stem Cell Educator therapy (*P* = 0.015, Figure 5A). Additionally, participants exhibited significant up-regulation of Th2 cytokines IL-4 and IL-5 expression at the 4-week follow-up (*P* = 0.012 and *P* = 0.022, respectively, Figure 5A). Expression of IL-13 was also significantly increased in 6/9 participants (3.69 ± 3.27 versus 15.55 ± 7.48, *P* = 0.005). No changes were observed in the level of Th1 cytokine IL-12 (*P* = 0.24, Figure 5A). Thus, these data suggested that the up-regulation of Th2 cell responses may suppress the Th1 cell-mediated autoimmune response in AA subjects [1,4,31] via associated cytokines [32]. The Stem Cell Educator therapy may shift the balance towards Th2-mediated immune responses, leading to the clinical efficacy in AA subjects.

The costimulatory molecule, CD28, functions as a key signal leading to Th2 cell differentiation and activation [33-36]. To determine changes in expression of costimulatory molecules, we examined lymphocytes for their levels of CD28 and inducible costimulator (ICOS). Flow cytometry demonstrated that the expression of CD28 was markedly increased in 8/9 participants 4 weeks after Stem Cell Educator therapy (*P* = 0.013, Figure 5B), but levels of ICOS were unchanged in all participants (*P* = 0.84, Figure 5B). Therefore, the up-regulation of CD28 expression, together with the increase of IL-4 production in AA subjects, can provide critical signals that shift the differentiation of human CD4^+^ T cells into Th2 cells [36] and attenuate Th1 cell responses.

### Formation of a “ring of TGF-β1” leading to local immune modulation and restoration of immune privilege of hair follicles after Stem Cell Educator therapy

To clarify the molecular and cellular mechanism underlying the regrowth of hairs and the immune modulation, we performed immunohistochemistry on fresh tissues via the biopsy of alopecic lesions from subjects after receiving Stem Cell Educator therapy. The histology of the alopecic lesions demonstrated a dense, perifollicular lymphocytic infiltration around anagen hair follicles. Twelve weeks after receiving Stem Cell Educator therapy, histological examination revealed the restoration of hair follicle architecture and the disappearance of the substantial perifollicular infiltration of lymphocytes. Additionally, horizontal sections confirmed an increase in the density of total hair follicles in these participants with alopecia totalis.

TGF-β1 is a pleiotropic growth factor that plays a key role not only in the induction of local immune tolerance [29], but also in the regulation of cycling of hair follicles via the inhibition of keratinocyte proliferation and the induction of apoptosis [30,37,38]. Previous works demonstrated that TGF-β1 contributed to the therapeutic efficacy of Stem Cell Educator therapy in both autoimmune-caused diabetic NOD mice [23] and diabetic patients [24,25]. Notably, in addition to the up-regulation of TGF-β1 expression in the peripheral blood mononuclear cells (Figure 5A), immunohistochemistry demonstrated that the level of TGF-β1 expression post-treatment (21.78 ± 0.27) was much higher than that of baseline before treatment (5.14 ± 0.01, *P* = 3.14337E-14), specifically at the proximal anagen hair follicle (Figure 5C, middle panel). The expression of TGF-β1 formed a cycle around the hair follicles (Figure 5C, bottom panel), similar to the “ring of TGF-β1” that we observed in a previous study [23]. It suggested that this unique feature of TGF-β1 distribution may contribute to reconstitution of the immune privilege of hair follicles and protect the regenerated hair follicles against further destruction by autoimmune cells. HE staining further demonstrated the reduction of immune cell infiltration and restoration of normal architecture of the hair follicles (Figure 5D).

To elucidate the major origin of the TGF-β1 production involved in the formation of the “ring of TGF-β1,” we performed double immunostaining in biopsied tissues by using the markers of monocyte/macrophage (CD14 and CD11b), Langerhans/dendritic cells (CD83 and DEC205), myeloid dendritic cells (CD1c), and Treg FoxP3, respectively. There were a few CD14^+^ monocytes/CD11b^+^ macrophages, Langerhans/dendritic cells (positive with CD83, DEC 205, or CD1c), and FoxP3^+^ Treg cells distributed in the dermal tissues. Double immunostaining showed weak staining for TGF-β1 in these immune cells, indicating that these immune cells were not the major source of TGF-β1. Notably, we found that there was a strong expression of TGF-β1 in the proximal root sheath of hair follicles of participants after receiving Stem Cell Educator therapy (Figure 5C, bottom panels) relative to the baseline level (Figure 5C, top panel). These data suggested that keratinocytes were recovered after receiving Stem Cell Educator therapy and became the major TGF-β1-producing cells leading to the restoration of immune privilege and the induction of immune tolerance in hair follicles. The molecular mechanisms underlying the up-regulation of TGF-β1 in keratinocytes need to be clarified in future studies.

## Discussion

AA is a devastating autoimmune disease that affects patients’ daily lives. Immune dysfunction of AA subjects is complicated, not only localizing in hair follicles, but also having effects outside of hair follicles with the development of other autoimmune diseases. Overcoming the autoimmunity represents one of the key hurdles in the treatment of AA. Systematic applications of immunosuppressive regimens usually yield significant side effects. Localized therapies have been widely utilized in clinic, including intralesional injections of glucocorticoids and the use of topical sensitizers through the induction of contact allergy (for example, dithranol and diphenylcyclopropenone), as well as topical corticosteroids and minoxidil [1,3,39]. To date, although a multitude of therapeutic options exist, neither local treatments nor systematic approaches can provide a cure for AA subjects [1,39]. Current clinical proof-of-concept data reveal the safety and efficacy of the Stem Cell Educator therapy approach in the treatment of AA subjects, as demonstrated by clinical outcomes in hair regrowth. This finding opens up a new avenue for AA clinical treatment by using the comprehensive immune modulation induced by Stem Cell Educator therapy. Because this disorder affects children of all hair colors [1], it is important to highlight that pediatric apheresis presents unique challenges due to children’s low body weight (<40 kg) and height (<140 cm) and the difficulties in vascular access and clinical monitoring. To overcome these technical hurdles and improve the safety in pediatric apheresis, alternative approaches should be considered for the treatment of pediatric AA subjects such as the application of a different apheresis machine with low extracorporeal blood volume, blood priming, and femoral vein catheterization.

AA is characterized as a T cell-mediated autoimmune disease, and CD8^+^ T cells seem to dominate the response. They may recognize the MHC class I-restricted melanogenesis-associated autoantigens and/or anagen-associated hair follicle autoantigens and thereby mediate the destruction of hair follicles [1,14]. More recently, CD8β^+^NKG2D^+^ T cells have been characterized as a major player leading to the autoimmune destruction of hair follicles [27]. Therefore, it is essential to attenuate these effector CD8^+^ T cells through the induction of peripheral immune tolerance to these self-antigens. Notably, we found that co-culture with CB-SCs could suppress the proliferation of activated CD8β^+^NKG2D^+^ T cells and reduce their percentage. Up-regulation of the expression of coinhibitory molecules BTLA and PD-1 on CD8β^+^NKG2D^+^ T cells may further attenuate their cytotoxic effects. The current study confirmed the expression of BTLA ligand HVEM on CB-SCs. Previous work demonstrated that the strong expression of programmed death-1 ligand (PD-L1) on CB-SCs contributed to the immune modulation of CD8 T cells [28]. Thus, CB-SCs may directly modulate CD8β^+^NKG2D^+^ T cells through the PD-1/PD-L1 and BTLA/HVEM pathways.

Additionally, CB-SCs strongly express the autoimmune regulator (Aire) [24] transcription factor. Aire proteins are usually found in thymic medullary epithelial cells, which play a central role in T cell development and the induction of immune tolerance by mediating ectopic expression of peripheral self-antigens and mediating the deletion of autoreactive T cells [40,41]. Knockdown of Aire protein expression resulted in a reduction of PD-L1 expression on CB-SCs. Thus, we hypothesize that, in a way, the Stem Cell Educator therapy may function as “an artificial thymus” that circulates a patient’s blood through a blood cell separator [20], briefly allows interactions between T cells and other immune cells with CB-SCs *in vitro*, induces immune tolerance through the actions of Aire [24], expression of PD-L1 and HVEM, the release of soluble factors (nitric oxide and TGF-β1), and cell-cell contacting mechanisms [20,28], returns the educated autologous lymphocytes to the patient’s circulation, and achieves immune balance and homeostasis in these AA subjects. Of interest, apheresis only withdraws approximately 10-15% of all lymphocytes, and since many pathogenic immune cells likely remain in the hair follicles and the connective tissue sheath which fail to enter into the blood circulation during the apheresis procedure, many autoimmune cells escape direct interaction with the CB-SCs. This would suggest that some of the cells altered by direct encounter with CB-SC can spread the tolerance systemically. Additionally, due to the short life span of most lymphocytes, subjects with severe AA may need additional treatments, perhaps at 3- to 6-month intervals, to improve the efficacy and possibly prevent the relapse of disease. To improve the clinical efficacy of Stem Cell Educator therapy, the sooner treatment with this therapy begins after diagnosis, the higher the chance of rescuing hair follicles and finding a cure for AA.

Animal and clinical studies demonstrated that both CD4^+^ Th1 cells and CD8^+^ T cells are required for the pathogenesis of AA [1,4,42]. Up-regulation of Th1 cytokines in AA subjects, not Th2 cytokines, exacerbates the autoimmune destruction [31,43,44]. Kubo and colleagues reported that there was a positive correlation between the severity of the alopecia and the increase of Th1 cells, inversely proportional to the number of IL-4-producing Th2 cells [45]. Therefore, the promotion of Th2 immune responses has been proposed to be beneficial for the treatment of AA patients [1,4]. The current study demonstrated that Th2 response-associated cytokines such as IL-4, IL-5, and IL-13 in these AA subjects were markedly increased after receiving Stem Cell Educator therapy. Additionally, CD28, one of the major costimulatory molecules contributing to the differentiation of Th2 cells [33-36], was up-regulated after Stem Cell Educator therapy. Thus, the combination of CD28 + IL-4 can provide key signals facilitating naïve or memory CD4^+^ T cells and giving rise to Th2 cells via the activation of mitogen-activated protein (MAP) kinase and extracellular signal-regulated kinase (ERK) signaling pathways [36]. Consequently, these Th2 cells and their cytokines may antagonize Th1 cell functions and counterbalance their AA-related autoimmune responses [32].

Collapse of immune privilege in hair follicles is the major cause of pathogenesis in AA. Due to constitutively low or absent expression of MHC class I antigen in the proximal hair follicle epithelium, hair follicles may initially be attacked by NK cells through NK cell function-activating receptors NKG2D and NKG2C [5,46]. Thus, attenuating NK cells is also necessary to reestablish the immune privilege and fundamentally advance the clinical outcomes for the treatment of AA. Notably, current data provide evidence for the up-regulation of TGF-β1 production in peripheral blood mononuclear cells, as well as the formation of a “ring of TGF-β1” in hair follicles of AA subjects after receiving Stem Cell Educator therapy. TGF-β1 can significantly suppress the proliferation and activity of NK cells [47], in addition to its effects on CD4^+^ and CD8^+^ T cells [29]. Thus, TGF-β1 may play a key role in the restoration of immune privilege of hair follicles and the induction of local immune tolerance.

It is also well known that TGF-β1 is a pleiotropic growth factor that plays a key role in the production and remodeling of the extracellular matrix [48]. Animal studies show that TGF-β1 acts as an essential factor contributing to the regulation of cycling and remodeling of hair follicles via the inhibition of keratinocyte proliferation and induction of apoptosis [30,37,38], as well as one of the key niche factors that regulate melanocyte stem cell immaturity and quiescence in the bulge area of hair follicles [49]. Previous work demonstrated that the formation of a “ring of TGF-β1” around pancreatic islets may protect the newly regenerated islet β cells against infiltrating lymphocytes and macrophages [23], providing a safe environment for promotion of regeneration of pancreatic islet β cells in long-standing type 1 diabetic patients [20,24]. Thus, the formation of a “ring of TGF-β1” may not only protect hair follicles through the restoration of immune privilege, but may also lead to the activation of epithelial hair follicle stem cells and hair regrowth. Additional molecular and cellular mechanisms underlying the Stem Cell Educator therapy in humans can be further explored by studying easily accessible and abundant hair follicles. Thus, clinical success in AA by the Stem Cell Educator therapy approach may open up new avenues for the treatment of other autoimmune diseases.

## Conclusions

AA is one of the most common skin autoimmune diseases, significantly affecting the life quality of patients. The current phase 1/phase 2 study demonstrates the safety and feasibility of Stem Cell Educator therapy in the treatment of AA subjects. Findings from this trial provide visible evidence that Stem Cell Educator therapy can control the autoimmunity and lead to hair regrowth.

## Abbreviations

AA: alopecia areata
AIRE: autoimmune regulator
BTLA: B and T lymphocyte attenuator
CB-SC: cord blood-derived multipotent stem cell
CD: cluster differentiation antigen
CFSE: carboxyfluorescein succinimidyl ester
DC: dendritic cell
ICOS: inducible costimulator
IL-4: interleukin-4
IL-5: interleukin-5
IL-12: interleukin-12
IL-13: interleukin-13
NOD: non-obese diabetes
PD-1: programmed death-1 receptor
TGF-β1: transforming growth factor β1
Th: helper T cells
Treg: regulatory T cells
